# Sex, cells, and metabolism: Androgens temper Th17-mediated immunity

**DOI:** 10.1172/JCI186520

**Published:** 2024-12-02

**Authors:** Nikita L. Mani, Samuel E. Weinberg

**Affiliations:** 1 Department of Pathology and; 2 Center for Human Immunobiology, Northwestern University Feinberg School of Medicine, Chicago, Illinois, USA.

## Abstract

Sex-based differences in autoimmune disease susceptibility have long been recognized, prompting investigations into how sex hormones influence immunity. Recent advances suggest that hormones may shape immune responses by altering cellular metabolism. In this issue of the *JCI*, Chowdhury et al. authenticates this model, showing that androgen receptor signaling modulates T helper 17 (Th17) cell metabolism, specifically glutaminolysis, reducing airway inflammation in males. This work provides insight into sex-specific regulation of immunity, highlighting the interplay between hormones, metabolism, and immune function. The findings raise intriguing questions about how hormonal fluctuations affect immunity and how sex-specific metabolic pathways might be leveraged for targeted therapies in autoimmune diseases.

## Linking autoimmune sex differences to immune cell metabolism

Biological sex is a major risk factor in the development of autoimmune diseases, with striking disparities observed between males and females (1). While most autoimmune conditions, including systemic lupus erythematosus, rheumatoid arthritis, and multiple sclerosis, show a strong female predominance, others, such as ankylosing spondylitis and some vasculitides, are more common in males (2). This complex landscape of sex-biased disease susceptibility underscores the profound influence of biological sex on immune function (3). Despite extensive research, the precise physiological mechanisms driving these differences remain largely elusive, with proposed factors ranging from genetics and hormones to environmental influences (1–4).

An emerging area of investigation at the intersection of biological sex and immunity is cellular metabolism. The field of immunometabolism has demonstrated that immune cell fate and function are intimately tied to the cellular metabolic states (5). Changes in nutrient utilization, energy production, and metabolic flux can profoundly impact immune cell differentiation, proliferation, and effector activity (6). Importantly, fundamental metabolic processes differ between males and females, with sex hormones exerting substantial influence over bioenergetic and biosynthetic pathways (7, 8). This sex-specific metabolic programming extends to immune cells, potentially shaping their development and effector functions in sex-dependent ways (9). Unraveling the complex interplay between sex hormones, metabolism, and immune function, alongside other physiological factors like diet, represents a critical frontier in immunology with important implications for understanding sex differences in immune responses and their impact on health and disease.

In this issue of the *JCI*, Chowdhury et al. address this knowledge gap head on, investigating how biological sex shapes T cell metabolism and function in the context of allergic airway inflammation (10). The study offers compelling evidence that androgen signaling fundamentally alters T helper 17 (Th17) cell metabolism, providing mechanistic insight into sex differences in allergic responses and potentially broader implications for autoimmune disease susceptibility.

## T cell–intrinsic androgen receptor signaling attenuates allergic airway inflammation

Chowdhury and colleagues began their investigation by exploring the role of androgen receptor (AR) signaling in allergic airway inflammation (10). Building on previous research establishing the importance of AR signaling in Th17-mediated inflammation (11–13), the authors set out to causally test the role of AR in a widely-used house dust mite (HDM) model of allergic inflammation that mimics human allergic asthma. Interestingly, the authors found that T cell–specific genetic deletion of AR increased HDM-induced disease severity in male mice to levels comparable to the heightened level observed in WT females. Importantly, organism-wide pharmacological blockade of AR recapitulated this finding. Both interventions resulted in elevated levels of inflammatory cell infiltration, airway hypersensitivity, and proinflammatory cytokines, including increased IL-17, in AR signaling–deficient male mice. Importantly, lack of AR signaling in female T cells had no impact on disease severity. These findings suggest that AR signaling in T cells is a primary driver of the reduced severity of HDM-induced inflammation observed in male mice, providing compelling evidence for the role of androgens in modulating allergic airway inflammation (10).

## Androgen-mediated suppression of glutamine metabolism regulates Th17 cell function

Next, the authors sought to determine if altered metabolism could explain the increased inflammation observed in male mice lacking AR signaling in T cells. Their initial analysis of T cell subsets from lung-draining lymph nodes revealed elevated levels of glutamate dehydrogenase 1 (GLUD1) in females compared with males, suggesting a potential sex-specific difference in glutamine metabolism. Further analysis of Th17 cells from AR-deficient male mice confirmed elevated levels of GLUD1, comparable to levels seen in female controls. Subsequent transcriptomic and metabolomic analyses identified upregulated glutamine metabolism in these cells, with enhanced glutamine anaplerosis into the TCA cycle. These findings implicate altered glutamine metabolism as a potential driver of enhanced Th17 cell activity in the absence of AR signaling (10).

To directly link AR signaling to altered glutamine metabolism and Th17 cell function, Chowdhury et al. (10) generated mice lacking glutaminase (GLS), specifically in T cells. When challenged in the HDM model, the results were striking. Male mice that underwent gonadectomy (removing androgen production) and female mice both showed decreased disease severity and reduced Th17 cell numbers compared with their respective controls when GLS was absent in T cells. In contrast, androgen-producing males showed no change when GLS was deleted from T cells. Supporting these findings, the authors also performed in vivo CRISPR screening, which identified multiple genes involved in glutamine metabolism as drivers of Th17 cell proliferation and fitness in gonadectomized male and WT female mice.

**Figure 1:**
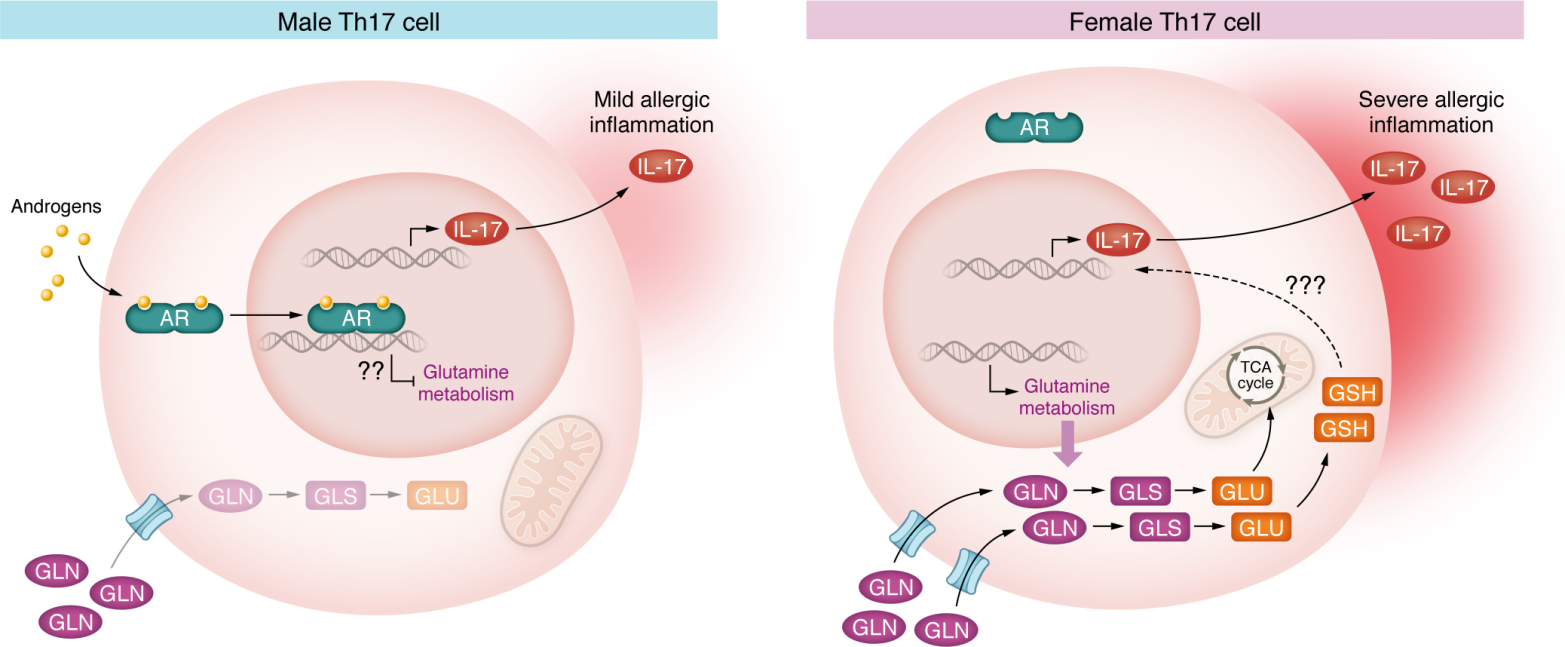
Androgen receptor signaling drives differences in Th17 cell metabolism and function during allergic airway inflammation. In male Th17 cells, androgen receptor (AR) signaling suppresses glutamine metabolism, resulting in mild allergic inflammation and reduced IL-17 production. The presence of androgens activates AR, which translocates to the nucleus and potentially regulates genes involved in glutamine metabolism, though the exact mechanism of AR-mediated suppression of glutamine metabolism remains to be fully elucidated. In contrast, female Th17 cells exhibit enhanced glutamine metabolism and severe allergic inflammation. The absence of AR signaling allows for increased glutamine (GLN) uptake through plasma membrane transporters and elevated glutaminase (GLS) activity. This leads to higher glutamate (Glu) production, which feeds into the TCA cycle and contributes to glutathione (GSH) synthesis. The enhanced glutamine metabolism in female Th17 cells correlates with increased IL-17 production and secretion, exacerbating allergic inflammation. Further research is needed to elucidate the precise molecular mechanisms linking enhanced glutamine metabolism to increased IL-17 production and Th17 cell pathogenicity in females, as the exact pathway has yet to be fully characterized.

These findings collectively demonstrate that androgen signaling fundamentally alters the metabolic state and functional capacity of Th17 cells during allergic airway inflammation. In the presence of androgens, male Th17 cells display reduced glutamine metabolism and inflammatory activity. However, in the absence of androgen signaling — whether in females or in androgen-depleted males — Th17 cells engage glutamine metabolism, which is required for their enhanced inflammatory potential (Figure 1). This AR signaling–mediated metabolic reprogramming of Th17 cells provides a mechanistic basis for the sex-specific differences observed in allergic airway inflammation, highlighting a critical role for glutamine metabolism in modulating immune responses in a hormone-dependent manner.

Finally, to validate these findings in a clinical context, Chowdhury et al. (10) examined T cells from patients with asthma. Consistent with their mouse models, Th17 cells from males with asthma showed reduced glutamine uptake compared with those from female patients. This observation in human samples underscores the link between androgen signaling, glutamine metabolism, and Th17 cell function in allergic airway inflammation.

## Implications and future directions

The study by Chowdhury and colleagues provides compelling evidence that AR activity plays a critical role in regulating Th17 cell metabolism and function in allergic airway inflammation (10). Their findings demonstrate that androgen signaling limits glutaminolysis in Th17 cells in males, resulting in reduced airway inflammation. While these insights are crucial, they also highlight the need to explore the broader implications of hormone-mediated metabolic regulation in immune cells across different contexts. The complexity of Th17 cell biology and its relationship with sex hormones extends beyond the lung inflammation model used in this study, as evidenced by the varied sex-based prevalence of Th17-associated autoimmune diseases. The variability in sex bias may be partly explained by the diverse roles and metabolic status of Th17 cells in different tissues. For instance, prior research has identified metabolically distinct pathogenic and protective Th17 cells in the intestines, demonstrating that metabolism may differ based on Th17 tissue context and function (14). Interestingly, the incidence of Crohn’s disease, thought to be driven in part by Th17 cell–mediated inflammation, increases in women following puberty, suggesting a potential role for estrogens in modulating disease risk (15). This observation, coupled with Chowdhury and authors’ (10) data showing that loss of estrogen receptor on T cells reduces disease severity in females, underscores the complex and sometimes opposing roles that different sex hormones can play in immune regulation. These findings highlight the need for a nuanced understanding of how androgens, estrogens, and other sex-specific hormones interact to shape Th17 cell function and immune responses across various tissues and disease contexts.

While the study by Chowdhury et al. (10) provides crucial insights into the role of androgen signaling in Th17 cell function, it also raises intriguing questions about the broader landscape of Th17 cell metabolism. Previous research has established that cell metabolism plays a critical role in Th17 cell differentiation and function (14, 16), with the balance of glycolysis and oxidative phosphorylation being particularly important for their inflammatory activity (17–19). This study breaks new ground by highlighting the regulation of glutamine metabolism by sex hormones in Th17 cells (10). However, mechanistic questions remain about how precisely glutamine metabolism enhances Th17 cell function. Glutamine serves multiple functions in T cells, including fueling the TCA cycle, contributing to glutathione synthesis, and generating α-ketoglutarate, which acts as a signaling molecule influencing metabolic adaptations and gene expression. Intriguingly, the authors demonstrate that the level of reactive oxygen species (ROS) in male Th17 cells is sensitive to AR activity, and loss of AR signaling in male Th17 cells increases glutamine flux to glutathione. These observations support a potential link between AR signaling, glutamine metabolism, oxidative stress, and Th17 cell development and function, as proposed by other recent studies (20, 21). However, more work is needed to fully elucidate this mechanism and its implications for Th17-mediated immune responses and related diseases.

In summary, the work of Chowdhury et al. (10) illuminates the complex interplay between androgen signaling, Th17 cell metabolism, and immune function, while also opening avenues for future research. Key questions remain: How do hormonal fluctuations across different physiological states affect Th17 metabolism and immune responses? Can we leverage our understanding of sex-specific metabolic pathways to design more effective therapeutic interventions? Future studies should explore these sex differences in Th17 metabolism across various organs and disease states, potentially leading to targeted therapies that account for the intricate relationship between sex hormones, cellular metabolism, and immune function. Such research could markedly advance our approach to treating mediated autoimmune and inflammatory diseases.

## Version 1 12/02/2024

Electronic publication
